# Systemic treatment options for advanced biliary tract carcinoma

**DOI:** 10.1007/s00535-020-01712-9

**Published:** 2020-08-03

**Authors:** Changqing Xie, Nicole A. McGrath, Cecilia Monge Bonilla, Jianyang Fu

**Affiliations:** Thoracic and Gastrointestinal Malignancies Branch, Center for Cancer Research, National Cancer Institute, National Institutes of Health, Bethesda, MD USA

**Keywords:** Biliary tract cancer, Chemotherapy, Targeted therapy, Immunotherapy

## Abstract

Advanced biliary tract cancers (BTC) include a diverse collection of rare and heterogenous tumors with poor prognosis. The combination of gemcitabine and cisplatin is the established first-line therapy for advanced BTC. There are no accepted standard treatments in the second line setting, though there are several ongoing clinical trials that implement chemotherapy as a therapeutic strategy. The understanding of the molecular landscape of BTC has offered hope of targeted therapies to the identified actionable genomic aberrations, such as FGFR2 gene fusions, mutations of IDH1/2, HER2, BRAC1/2 and BRAF. Pembigatinib has become the first approved targeted therapy for BTC with FGFR2 fusion or other rearrangements. Recent immunotherapy has opened new therapy avenues in BTC with pembrolizumab approved for either microsatellite instability high (MSI-H) or DNA mismatch repair deficient (dMMR) advanced solid tumors, including BTC. The combination of immunotherapy with other modalities is currently being evaluated in different clinical trials, since single agent immunotherapy appears to provide modest benefits in advanced BTC. In this review, we summarize the current status of treatment options, including systemic chemotherapy, targeted therapy, immunotherapy, and various combinations in advanced BTC.

## Introduction

Biliary tract carcinoma (BTC) typically refers to malignancies originating in the intra- and extrahepatic biliary ductal system, known as cholangiocarcinoma (CCA), and the gallbladder; however, periampullary tumors are also included as BTCs. The primary risk factors for intrahepatic cholangiocarcinoma (iCCA) and extrahepatic cholangiocarcinoma (eCCA) include chronic inflammatory diseases of the biliary tract such as primary sclerosing cholangitis and hepatolithiasis, congenital hepatobiliary anomalies, liver fluke infection, non-alcoholic fatty liver disease [1–4]. Moreover, cholelithiasis with the presence of chronic inflammation is the most prevalent risk factor for gallbladder cancer (GBC) [5]. The anatomic subtypes of BTC display distinguished molecular aberrations [6], that suggests complexities of pathogenesis of BTC. Commonly occurring genetic aberrations in iCCA include TP53 (35%), KRAS (24%), ARID1A (20%), IDH1 (18%), MCL1 (16%), and PBRM1 (11%). In contrast, the most frequent aberrations in eCCA are TP53 (45%), KRAS (40%), ERBB2 (25%), SMAD4 (25%), FBXW7 (15%) and CDKN2A (15%) [7]. The clinical presentation of BTC differs with their sites, that patients with eCCA usually present with symptoms and painless jaundice is the most common, while the most common presentation of iCCA and GBC is of incidental discovery [1].

Although BTC is a relatively uncommon diagnosis, studies have suggested that the incidence and mortality of BTC has increased in recent years [8–10]. Among new cases of BTC that are diagnosed each year in the United States, there are approximately 3,000 cases of intrahepatic cholangiocarcinoma (iCCA), 11,000 cases of extrahepatic cholangiocarcinoma (eCCA) and gallbladder carcinoma (GBC) [11]. Possibly due to these location related mutations and the ease of total resection, patients diagnosed with distal eCCA tend to have better outcomes than patients with perihilar eCCA and iCCA [12]. The current 5-year survival rate in patients with early stage BTC who undergo curative-intent surgery is 30% [13]. Patients with advanced disease at diagnosis have limited treatment options and poor prognosis. This review is focused on systemic therapy options for advanced BTC, including chemotherapy, targeted therapy, and immunotherapy.

### Chemotherapy

Systemic chemotherapy is the standard of care for the patients with advanced BTC. There is evidence from several trials to support the use of gemcitabine-based or fluoropyrimidine-based chemotherapy for patients with advanced BTC [14–17]. Among these, a randomized, multicenter, phase III study in advanced BTC evaluated overall survival (OS) with the combination of gemcitabine/cisplatin (GemCis) in comparison to gemcitabine alone (ABC-02) [18]. In total, 410 patients with locally advanced and metastatic BTC were enrolled and randomly assigned to a treatment. The results showed that the addition of cisplatin significantly improved progression-free survival (PFS) and OS by 3 months. The median PFS and OS were 8 and 11.7 months, respectively, with a median follow-up period of 8.2 months in the GemCis cohort. The tumor control rate was 81.4% in the GemCis cohort, compared to 71.8% in treatment with gemcitabine alone [18]. This study established GemCis as the current first line of care chemotherapy in advanced BTC, with subsequent studies further validating their results.

For example, a similar regimen was tested in a multicenter, randomized, phase II study of advanced BTC in Japan (BT22). Among 83 patients analyzed, the overall response rate (ORR) was 19.5% in the GemCis combination cohort versus 11.9% in the gemcitabine treatment group. Adding cisplatin to the treatment regimen increased OS and PFS by 2 and 3 months, respectively [16]. Overall, the combination was safe and adverse events were similar in the two groups, with the exception of a higher incidence of neutropenia in the GemCis group [16, 18]. Subsequent additions to gemcitabine/platinum with targeted therapeutic agents have failed to demonstrate better outcomes to date, with a range of median OS of 7.7-14.1 months [17, 19–21]. A recent report from a phase III randomized trial have posited that the combination of gemcitabine plus oral fluoropyrimidine S-1 showed non-inferior to GemCis in terms of OS and PFS in comparison to GemCis regimen and well tolerated among recruited 354 patients, although there were schedule difference of GemCis used in this study and those in the original ABC studies [22]. There are ongoing trials utilizing the strategies with combinations of targeted therapy and chemotherapy with the hope of new first line of care options for patients with advanced unresectable BTC (Table 1). Unfortunately, no randomized phase III trials with such combinations are yet in progress.

**Table 1:** Ongoing trials of chemotherapy with or without targeted therapy in advanced BTC

Regimen	Phase	Trial ID	Design
Trifluridine/Tipiracil/Irinotecan	II	NCT04059562	AC
Trifluridine/Tipiracil	II	NCT04076761	AC
FOLFIRINOX	II	NCT04305288	AC
FORFIRINOX	II	NCT03772132	BD
Nal-IRI/5-FU/leucovorin	II	NCT03043547	AC
Irinotecan/Trifluridine and Tipiracil	II	NCT04072445	AC
Nal-Irinotecan/Trifluridine and Tipiracil	I/II	NCT03368963	AC
Chemotherapy/EphB4-HAS/	I	NCT02495896	AC
GemCis + Copanlisib	II	NCT02631590	AC
GemCis/Anetumab ravtansine	I	NCT03102320	BD
FORFIRINOX/target agents	II	NCT03768375	BD
GEMOX/target agents	II	NCT02836847	BD
GemCis/Ivosidenib/Pemigatinib	I	NCT04088188	BD
GemCis/CPI 613	I/II	NCT04203160	AC
GemCis/CX-4945	I/II	NCT02128282	AC
GemCis/Pemigatinib	I/II	NCT02393248	BD
Carboplatin/Paclitaxel/Pevonedistat	II	NCT04175912	AC
GemCis/FT-2102	I/II	NCT03684811	BD

*FORFIRINOX* folinic acid, fluorouracil, irinotecan, oxaliplatin, *nal*-*IRI* liposomal irinotecan, *GemCis* gemcitabine cisplatin, *GEMOX* gemcitabine oxaliplatin, *AC* all-comers, *BD* biomarker driven

More intensive, triple chemotherapy regimens have been evaluated and have proven to be effective in a small number of patients with advanced BTC. A randomized phase III trial was conducted to evaluate the combination of epirubicin, cisplatin and 5FU (ECF) versus 5FU, etoposide and leucovorin (FELV) in patients with advanced BTC as a first line of treatment. The results from enrollment of 54 patients showed no difference between two groups in terms of ORR and median OS for ECF and FELV were 9.02 months and 12.03 months, respectively; however, no significant difference was detected between the groups [23]. In a more recent phase II trial of advanced BTC, patients were treated with the addition of 5-FU to GemCis (GFP). There were 21 patients enrolled and impressive anti-tumoral activity was exhibited by a median OS of 18.8 months and a median time to progression of 13.4 months [24]. Additionally, fifty patients with advanced BTC in the KHBO 1002 phase II trial were treated with GemCis/S-1 and showed a median OS of 16.1 months [25]. The phase III trial of this combination has been completed and authors reported similar findings to their phase II trial (KHBO1401-MITSUBA) [26]. Finally, the combination of GemCis/nab-paclitaxel was tested in a phase II trial and 60 patients with metastatic or unresectable BTC showed a median PFS of 11.4 months and median OS of 19.2 months [27].

There is no recommendation for a second-line therapy; however, numerous of trials with various chemotherapy combinations have been conducted on the subject. A systematic review of second-line chemotherapies in advanced BTC has been conducted, which included 25 studies (14 phase II clinical trials, 9 retrospective analyses, and 2 case reports) and the outcomes from 761 patients were reported. The mean PFS, OS, and response rate were 3.2 months, 7.2 months, and 7.7%, respectively [28]. ABC-06 is an open-label, randomized, multicenter study comparing FOLFOX (5-FU and oxaliplatin) as second line option with active symptom control after GemCis, that was recently completed (NCT01926236). The updated results showed that 81 patients in the FOLFOX cohort had a median OS of 6.2 months compared to 5.3 months in the cohort with active symptom control alone [29]. Though all of the trials had few patients, median OS ranged from 4 to 9 months, suggesting that treatment in the refractory setting is feasible and may provide a survival advantage when compared to active symptom control. Ongoing clinical trials utilizing chemotherapy to treat BTC are outlined in Table 1.

### Targeted therapies

The genetic landscape of BTC has been illuminated with the widespread use of next generation sequencing. More importantly, the understanding of this landscape has led to the identification of novel actionable drivers of BTC pathogenesis [3, 30] and rapid clinical trial development targeting various molecular aberrations (Table 2).

**Table 2:** Ongoing targeted therapy trials in advanced BTC

Target	Agent	Phase	Trial ID	Design
ATG7	AbGn-107	I	NCT02908451	BD
BRAF	ABM-1310	I	NCT04190628	BD
Cancer stemness kinase	BI503	II	NCT02232633	AC
CDK4/6	Abemaciclib	II	NCT03339843	AC
DNA polymerase	MIV-818	I/II	NCT03781934	AC
FGFR	E7090	II	NCT04238715	BD
FGFR	Pemigatinib	II	NCT02924376	BD
FGFR	Pemigatinib	II	NCT04256980	BD
FGFR	Infigratinib	II	NCT02150967	AC
FGFR	CPL304110	I	NCT04149691	BD
FGFR	Erdafitinib	II	NCT02699606	BD
FGFR	Infigratinib	II	NCT04233567	BD
FGFR	TAS-120	I/II	NCT02052778	BD
FGFR	HMPL-453	II	NCT04353375	BD
FGFR2	Derazantinib	II	NCT03230318	BD
FGFR4	INCB062079	I	NCT03144661	BD
HER2	Trastuzumab	II	NCT03613168	BD
HER2	A166	I/II	NCT03602079	BD
HER2	ZW49	I	NCT03821233	BD
HER2	DS-8201a	II	JMA-IIA00423	BD
IDH1	AG-120	III	NCT02989857	BD
IDH1	IDH305	I	NCT02381886	BD
IDH1	AG-120	I	NCT02073994	BD
Multi-kinase	EOC317	I	NCT03583125	BD
Notch	CB-103	I/II	NCT03422679	AC
PARP	Olaparib	II	NCT03212274	BD
PARP	Niraparib	II	NCT03207347	BD
Proteasome	Bortezomib	III	NCT03345303	AC
TRK	Entrectinib	II	NCT02568267	BD
VEGFR2	Regorafenib	II	NCT02162914	AC
VEGFR2	Apatinib	II	NCT03521219	AC
VEGFR-2	Ramucirumab	II	NCT02520141	AC
ATR/PARP	Ceralasertib/Olaparib	II	NCT03878095	BD

*AC* all-comers, *BD* biomarker driven

### FGFR inhibitors

Fibroblast growth factor receptor (FGFR) genetic alterations (fusions, amplifications, mutations) occur in 7–45% of iCCA cases, < 1–5% eCCA cases, and 3% GBC cases [30, 31]. Currently, many active clinical trials are enrolling patients to further evaluate the role of FGFR2 inhibitors in the management of BTC.

### Selective inhibitor

Pemigatinib (INCB054828) is a selective, oral inhibitor of FGFR 1, 2 and 3. A multicenter, open-label, single-arm, multicohort, phase II (FIGHT-202) study showed an ORR of 35.5% and disease control rate (DCR) of 82% in 107 patients with FGFR2 fusion or rearrangement. The median follow-up period was 17.8 months and the median PFS and OS were 6.9 and 21.1 months, respectively [32]. Hypophosphatemia, arthralgia, stomatitis, hyponatremia, abdominal pain, and fatigue were the most frequently reported adverse events. This trial has led to the FDA approval of pemigatinib for second line treatment options for patients with FGFR2 fusion or other FGFR2 rearrangement. Pemigatinib is the first approved targeted therapy for BTC. An open-labeled, randomized phase III study (FIGHT-302) was initiated in 2019 to evaluate pemigatinib as first line treatment compared to GemCis (NCT03656536).

Other selective FGFR inhibitors erdafitinib and rogaratinib were evaluated in BTC patients as part of basket trials and there was evidence of antitumor effects [33, 34]. Subsequently, erdafitinib was tested in a phase II study of 14 BTC patients with FGFR alterations. The study resulted in a DCR of 83.3% with a median PFS of 5.6 months [35]. Additionally, E7090 is a novel, selective FGFR inhibitor of FGFR1, 2, and 3, and is currently under first-in-human clinical evaluation (NCT02275910).

### Nonselective inhibitors

Infigratinib (BGJ398) is a nonselective pan-FGFR inhibitor and showed antitumor activity in a first-in-human phase I basket trial. Three advanced BTC patients (two had FGFR2 fusion and one with an FGFR2 mutation) exhibited stable disease with a 5–20% reduction in tumor size [36]. In a multi-institutional phase II trial, patients with advanced BTC who had FGFR genetic alterations (e.g., fusion, mutation, and amplification) demonstrated an ORR of 14.8% and a DCR of 75.4%, respectively. The median PFS was 5.8 months [37]. Hyperphosphatemia, fatigue, stomatitis, and alopecia were the most common adverse events reported. The drug is currently being evaluated in a multicenter, open-label, randomized phase III clinical trial involving patients with advanced BTC harboring FGFR2 gene fusions/translocations in the first line setting in comparison to GemCis (NCT03773302).

TAS-120, ponatinib and derazantibib are nonselective FGFR inhibitors. TAS-120 exhibited an ORR of 25.0% and a DCR of 78.6% in 28 patients with iCCA harboring FGFR2 fusion in a phase I basket study [38]. The phase II study is in progress [39]. In another phase 1 trial with a total of 19 BTC patients, TAS-120 demonstrated 50% ORR in patients with FGFR2 fusions and even additional clinical benefit for patients who have progressed on reversible FGFR inhibitors [40]. Ponatinib has shown efficacy in controlling disease in patients with advanced BTC harboring FGFR2 fusion [41]. In a recently completed phase II clinical trial, ponatinib was reported to achieve an ORR of 45%. The median PFS was 2.4 months and the median OS was 15.7 months (NCT02265341). Another basket trial with ponatinib is still ongoing for advanced solid tumors with an FGFR mutation (NCT02272998). Two of twelve iCCA patients with FGFR genetic alterations demonstrated a partial response in a phase I basket trial with derazantinib [42]. In a phase I/II trial, derazantinib’s ORR was 21%, DCR was 82.8%, and it exhibited a median PFS 5.7 months in 29 advanced iCCA patients with FGFR2 fusion [43]. Derazantinib is currently being tested in a phase II study for advanced iCCA and mixed hepatocellular carcinoma/BTC with FGFR gene aberrance after patients’ tumors have progressed with one or more systemic therapies (NCT03230318).

### IDH inhibitors

The incidence of isocitrate dehydrogenase 1/2 (IDH1/2) mutations is 4.9-36% in iCCA, 0-7.4% in eCCA, and 1.5% in GBC [30]. In general, IDH1 mutations are more common than IDH2 mutations. Pharmacologic inhibitors that are specific to IDH-mutations have been developed [44].

Ivosidenib (AG-120) is an oral, first-in-class inhibitor of mutant IDH1. The treatment efficacy of ivosidenib in BTC was examined in 73 advanced BTC patients with mutated IDH1. Among 72 evaluable patients, four (5%) patients had a partial response to the treatment. The median PFS and OS were 3.8 months and 13.8 months, respectively [45]. Common adverse events encountered included fatigue, nausea, diarrhea, abdominal pain, decreased appetite, and vomiting. Acquired resistance developed that was associated with a novel IDH2 mutation [46]. The ClarIDHy trial is a global, randomized, double-blind, phase 3 trial to evaluate ivosidenib efficacy in patients with advanced BTC with an IDH1 mutation in comparison to a placebo. In the trial, 185 patients with IDH1 mutated advanced BTC were enrolled and randomized in a 2:1 ratio of ivosidenib to placebo. The median PFS and OS were 2.7 months and 10.8 months with ivosidenib versus 1.4 months and 9.7 months with the placebo. The authors concluded that a favorable trend in OS was seen with ivosidenib treatment [47].

Enasidenib (AG-221) is a selective inhibitor of mutant IDH2 and is currently being assessed in multiple phases I/II clinical trials in subjects with advanced solid tumors, including IDH2 mutated BTC (NCT02273739). Other inhibitors of IDH1 (IDH305, NCT02381886) and pan-IDH1/2 (AG881, NCT02481154) are currently being tested in patients with BTC in phase I studies.

### EGFR inhibitor

EGFR is overexpressed in 8.1% of BTC [48], while KRAS mutations in BTCs were identified in 4-40% of cases [30]. Several EGFR targeted agents have been developed but the antitumor activity observed in BTC was modest. For example, the oral, reversible tyrosine kinase inhibitor (TKI) erlotinib was evaluated in advanced BTC and resulted in three partial responses [49]. When combined with gemcitabine/oxaliplatin (GEMOX) in a phase 3 trial [50], there was no difference in median OS or PFS; however, a significantly higher ORR was achieved in treatment with chemotherapy plus erlotinib when compared to GEMOX. Furthermore, of 49 evaluable patients treated with the addition of bevacizumab to erlotinib, six patients had partial responses and 25 maintained a stable disease. This combination resulted in a median OS 9.9 months [51]. Although non-randomized phase II trials showed the efficiency of cetuximab for advanced BTC in addition to chemotherapy [52–54], the randomized phase II trial (BINGO) with the combination of cetuximab with GEMOX failed to show OS benefit [20]. Two other randomized phase II trials did not find any considerable benefits of cetuximab and GEMOX or with the addition of capecitabine to GEMOX, even with stratification by KRAS mutation status [55, 56]. Panitumumab was evaluated in a phase II trial in combination with gemcitabine and irinotecan in patients with advanced BTC. The results showed an ORR of 39% and a median PFS and OS of 9.7 months and 12.9 months, respectively [57]. When panitumumab was combined with GEMOX followed by capcitabine treatment in KRAS wild type irresectable BTC, it showed an ORR of 46%. The median PFS and OS were 6.1 months and 9.5 months, respectively [58].

### VEGF inhibitors

VEGF is highly expressed in 40–75% of BTC [59]. Several antiangiogenic agents have been developed and tested in clinical trials of BTC; however, VEGF inhibition does not appear to be a relevant therapeutic target in BTC. For example, the combination of bevacizumab with GEMOX in 35 patients with advanced BTC was evaluated in a phase II study and the results showed an ORR of 40%, a median PFS of 7 months, and an OS of 12.7 months [60]. In the other phase II trial involved with a similar regimen, the addition of bevacizumab to GEMOX increased the PFS and but failed to provide an OS benefit [61]. The combination of FOLFIRI with bevacizumab resulted in an ORR of 38.4% and a median OS of 20 months [62]. Moreover, in a phase II trial to evaluate GemCis with or without cediranib (ABC-03), 124 patients with advanced BTC were enrolled. No significant improvements in either PFS or OS were observed; however, the addition of cediranib did improve the ORR [17]. Single agent sorafenib failed to show any clinical benefit with a reported median OS of 4.4 months [63], and 9 months in patients with advanced BTC [64]. No additional clinical benefit was observed when sorafenib was added to GemCis, yielding a median PFS and OS of 6.5 and 14.4 months, respectively. Furthermore, no significant differences were found when compared to historical controls using GemCis alone [65]. Moreover, a phase II clinical trial with regorafenib as a single agent treatment in advanced, refractory BTC exhibited a median PFS and OS was 2.2 and 4.5 months in 43 patients [66]. Sunitinib and Axitinib, were also evaluated in advanced BTC that was refractory to first line treatment [67–69] and vandetanib was evaluated alone or in combination with gemcitabine 70]. However, the role of these agents did not yield promising results.

### Tropomyosin receptor kinases (TRKs) inhibitor

Genomic translocations with chromosomal fusion lead to the constitutive activation of TRKs. Overexpression of TRKs in BTC has been reported but the incidence is variable, ranging from 0 to 20.5% [71–73]. Larotrectinib is a highly selective TRK inhibitor and was approved for solid tumors with NTRK gene fusion (NCT02122913, NCT02637687 and NCT02576431) [74]. Two patients with BTCs were included in the study, one achieving a nearly 80% decrease in initial tumor size. Moreover, adverse events were predominantly classified as grade 1. Entrectinib is a less selective inhibitor of TRK, targeting ROS1 and ALK. It has been evaluated in a phase 1 study of a selected population of solid tumors, including BTCs, and yielded an ORR of 57–86% [75]. Recent reports from three ongoing phase I/II clinical trials (ALKA-372-001, STARTRK-1, and STARTRK-2) showed an average ORR of 57% with one partial response in a BTC patient [76]. The most common treatment-related severe adverse events were weight gain, anemia, and neural system disorder.

### MET inhibitor

Overexpression of c-MET has been detected in 12-58% of iCCA [77, 78] and 16% of eCCA [77]. TKIs of the MEK pathway have also been studied in this disease. Carbozantinib is a TKI that targets MET and VEGFR2 and has demonstrated limited efficacy in a phase 2 trial, although, one patient with an MET-high tumor received a prolonged clinical benefit from treatment [79]. Tivantinib is an oral, selective c-MET inhibitor and was evaluated in combination with gemcitabine in patients with advanced solid tumors. The results showed that 20% of 56 patients had a partial response, including one patient with advanced BTC [80]. A non-selective MET inhibitor, merestinib, is currently being evaluated in combination with gemcitabine/cisplatin as a first-line of therapy (NCT02711553). Additionally, the results of a phase 2 trial evaluating Crizotinib, an ALK and c-Met inhibitor, for patients harboring an ALK, MET, or ROS1 alteration are pending completion of the trial (NCT02034981).

### MEK and BRAF inhibitors

The MEK1/2 inhibitor, selumetinib showed an ORR of 12% among 28 patients with metastatic BTC and median PFS and OS of 3.7 and 9.8 months, respectively [81]. Thirteen patients with advanced BTC were treated with selumetinib in combination with GemCis in a first-line setting (phase 1b trial, ABC-04). Patients had three partial responses and 5 maintained stable disease [82]. Selumetinib has continued onto evaluation in a phase 2 study with no results to date (NCT02151084).

Selective oral MEK1/2 inhibitor binimetinib exhibited two partial response and one complete response in BTC [83]. Enhanced antitumor effectiveness of binimetinib was observed in a phase 1b study when used in combination with capecitabine in the second-line setting [83]. The median PFS and OS were 3.9 months and 8.0 months, respectively. Additional evaluations of binimetinib in combination with GemCis in phase I/II studies showed an ORR of 36% in 35 advanced BTC patients with median PFS 6 months and an OS 13.3 months [84]. A study using an identical regimen in advanced BTC is ongoing (NCT02151084). In a randomized phase II study, trametinib monotherapy in patients with advanced BTC failed to show a significant clinical response when compared to the treatment of 5-FU combined with leucovorin or capecitabine [85]. Trametinib was also evaluated in a phase II study of patients with advanced BTC in a second-line setting and showed one partial response among 20 patients and a median PFS of 2.7 months and 1-year survival of 20% [86]. When combined with pazopanib on 25 patients with advanced BTCs, trametinib showed median PFS of 6.4 months [87].

Mutation of BRAF V600E is rare in BTC with a reported incidence of about 1-5%, moreover, typically confined to iCCA [70, 88]. In a phase II basket trial, only one patient out of 12 with iCCA demonstrated a partial response over a year of treatment with vemurafenib [89]. The combination of trametinib and dabrafenib was reported in case studies of advanced BTC with a BRAF V600E mutation and the results showed regression of metastatic lesions [90, 91]. In a basket trial of rare tumors, including BTC harbouring BRAF V600E mutations, treatment with dabrafenib and trametinib resulted in an ORR of 42% in 35 patients with BTC [92]. The median PFS and OS were 9.2 and 11.7 months, respectively.

### HER2/ERBB2 inhibitor

In BTCs, HER2 overexpression is observed in 5% of iCCA, 20% of eCCA, and 19% of GBC [93]. HER2 or ERBB2 amplification present in up to 20% of eCCA but is rare in iCCA [59, 94]. Alterations of the ERBB family have been reported in BTC, comprised of 19% of GBC, 17% of eCCA [7], and 4.8% of iCCA cases [93]. Single agent trastuzumab from a retrospective case series showed promising results on GBC but not in iCCA or eCCA with HER2/neu mutation [95]. The addition of chemotherapy to trastuzumab also showed clinical activity in the first line of care for refractory BTC [96]. Trastuzumab is being evaluated with GemCis as second line therapy in HER2-positive BTC in a phase II trial (NCT 03613168) and with GEMOX as a second-line therapy in advanced or recurrent eCCA and GBC (NCT02836847). A phase II trial on trastuzumab emtansine (TDM-1) in bladder cancer, pancreatic adenocarcinoma, and BTC was terminated before full enrollment (NCT02999672). The official results are pending but there was a 14% overall response of 7 patients from the cohort of pancreatic adenocarcinoma/BTC with median PFS of 2.5 months. Trastuzumab deruxtecan (DS-8201) is currently being evaluated in a phase II trial involved with HER2 positive BTC (JMA-IIA00423) [97]. Lapatinib, a dual TKI targeting EGFR and HER2, failed to show antitumor activity in advanced BTC in first and second-line setting as a single agent [98, 99]. An ongoing phase II basket trial (SUMMIT) is examining the efficacy of neratinib (TKI of EGFR, HER2, and HER4) in HER2-mutated cancers, including BTCs, and has shown a meaningful clinical response [100]. Interim results indicated an ORR of 10.5% among 10 enrolled patients with refractory BTC with 2 partial responses and 4 with stable disease. The median PFS was 1.8 months [101]. Varletinib (EGFR/Her2 co-inhibitor) has been evaluated with capecitabine in a randomized, controlled phase II/III trial (TreeTopp) in the second-line setting in BTC (NCT03093870) and with GemCis in first-line setting in advanced BTC (NCT02992340).

### PARP inhibitor

The presence of a germline mutation in BRCA1 or BRCA2 confers an increased lifetime risk of developing BTC [102] and somatic mutations of BRCA1 and BRCA2 have been reported in BTC [6]. BRCA-mutated tumors are often sensitive to poly [ADP-ribose] polymerase (PARP) inhibition [103]. In a retrospective clinical analysis in 18 patients with BRCA-mutated BTC, the median OS for patients, regardless of treatment modality with stage III/IV disease was 25 months, while 40.3 months with stage I/II stage, that appears longer than SEER historical control in general [104]. One of the four patients that received PARP inhibitors obtained a favorable disease response with a PFS duration of 42.6 months and an OS of 64.8 months [104]. A phase II trial of the PARP inhibitor niraparib is planned in patients with advanced-stage malignancies, including BTC, and with known mutations in BAP1 and other DNA double-strand break repair pathway genes (NCT03207347). There are studies of PARP inhibitors, Niraparib and olaparib ongoing in patients with BTC with aberrant gene mutations (NCT04042831, NCT03207347). A basket phase II trial of olaparib for patients with metastatic solid tumors with IDH1 or IDH2 mutations including BTCs has been undertaken (NCT03212274).

Other ongoing trials with molecular based targets in advanced BTC include copanlisib (PI3K inhibitor) (NCT02631590), ABC294640 (sphingosine kinase and JAK/STAT inhibitor) (NCT03377179, NCT03414489), ceritinib and crizotinib (ALK and ROS1 inhibitor) (NCT02374489, NCT02034981) (Table 2).

### Immunotherapy

Over the past two decades cancer treatment has seen much progress for immune-based approaches in solid tumor malignancies. Landmark FDA approvals for various strategies, including immune checkpoint inhibitor (ICI), targeting CTLA4 or the PD1/PDL1 axis have dramatically impacted the lives of patients. Presently, the clinical data on immunotherapy in BTC are limited to small single-arm studies and sub-analyses of basket trials [3, 105], though numerous clinical trials studying ICIs are underway (Tables 3, 4).

**Table 3:** Ongoing immunotherapy trials in advanced BTC

Target	Agent	Phase	Trial ID	Design
PD-1	Toripalimab (JS001)	I/II	NCT03867370	AC
PD-L1	M7824	II	NCT03833661	AC
PD-1	Pembrolizumab	II	NCT02628067	AC
PD-L1	STI-3031	II	NCT03999658	BD
CD166	CX-2009	I/II	NCT03149549	AC
PD-1/PD-L1	Pembrolizumab, nivolumab, atezolizumab, ipilimumab	III	NCT04157985	AC
PD-L1/CTLA-4	Durvalumab/Tremelimumab	II	NCT04238637	AC
CTLA-4, PD-1	Ipilimumab/Nivolumab	II	NCT02834013	BD
CTLA-4, PD-1	XmAb20717	I	NCT03517488	AC
CTLA-4, LAG3, PD-1	XmAb22841| Pembrolizumab	1	NCT03849469	AC
OX40, PD-1	ABBV-368| ABBV-181	1	NCT03071757	AC
CD40/CD135	CDX-1140| CDX-301| Pembrolizumab	1	NCT03329950	AC
TAA	TRK-950	1	NCT02990481	AC
Vaccine	Hepcortespenlisimut-L	I/II	NCT03042182	BD
	Hepcortespenlisimut-L	III	NCT02232490	BD
Cellular	MUC-1 CART cells	I/II	NCT03633773	BD
	Cytokine-induced killer cells	I/II	NCT01868490	AC
	Tumor Infiltrating Lymphocytes (TIL)	II	NCT03801083	AC
	Central memory T cells	II	NCT03820310	AC

*AC* all-comers, *BD* biomarker driven

**Table 4:** Ongoing trials of immunotherapy in combination with other in advanced BTC

Combination	Agent	Phase	Trial ID	Design
+ Immunotherapy	Durvalumab/CSF1R inhibitor	II	NCT04301778	AC
	Anti-PD-1/TC-210 T Cells	I/II	NCT03907852	BD
	Pembrolizumab/Allogeneic NK cell	I/II	NCT03937895	BD
	Pembrolizumab/Allogeneic T cells	I	NCT02757391	AC
	Rovalpituzumab Tesirine/ABBV181	I	NCT03000257	AC
	XmAb22841/Pembrolizumab	I	NCT03849469	AC
+ Immunotherapy +RT	Autologous DC/Pneumococcal Vaccine/RT	I	NCT03942328	AC
+ Chemotherapy	TRK950/GemCis	I	NCT03872947	AC
	Toripalimab/Gemcitabine/5-FU	II	NCT03982680	AC
	Durvalumab/GemCis	II	NCT04308174	AC
	Pembrolizumab/CapOx	II	NCT03111732	AC
	Pembrolizumab/GemCis	III	NCT04003636	AC
	Durvalumab/Tremelimumab/GemCis	II	NCT03473574	AC
	Bintrafusp alfa/GemCis	II/III	NCT04066491	AC
	Pembrolizumab/INT230-6	I/II	NCT03058289	AC
	Anti-CTLA-4/INT230-6	I/II	NCT03058289	AC
	Durvalumab/GemCis	III	NCT03875235	AC
+ Chemotherapy + Targeted therapy	JS001/GEMOX/Lenvatinib	II	NCT03951597	AC
+ Epigenetic modulator	Durvalumab/Guadecitabine	I	NCT03257761	AC
	Nivolumab/Entinostat	II	NCT03250273	AC
+ RT	Durvalumab/Tremelimumab/ablation	II	NCT02821754	AC
	Camrelizumab/Cryoablation	II	NCT04299581	AC
	Cytokine-induced killer cells/ablation	II/III	NCT02482454	AC
	Camrelizumab/Radiation	II	NCT03898895	AC
+ Targeted therapy	Pembrolizumab/Olaparib	II	NCT04306367	AC
	Atezolizumab/Cobimetinib	II	NCT03201458	AC
	LMB-100/Tofacitinib	I	NCT04034238	AC
	Toripalimab/Lenvatinib	II	NCT04211168	AC
	Durva/AZD6738/Olaparib	II	NCT04298021	AC
	Durva/AZD6738	II	NCT04298008	AC
	Nivo/FT2102	I/II	NCT03684811	BD
	Durvalumab/Olaparib	II	NCT03991832	BD
	Nivolumab/TPST-1120	I	NCT03829436	AC
	Pembrolizumab/Lenvatinib	II	NCT03895970	AC
	TQB2450/Anlotinib	I/II	NCT03996408	AC
	Anti-PD-1/IntegrinaV/b8 antagonist	I	NCT04152018	AC
+ Targeted therapy + RT	Avelumab/Nedisertib/Radiation	I/II	NCT04068194	AC

*GemCis* gemcitabine cisplatin, *GEMOX* gemcitabine, oxaliplatin, *CapOx* capecitabine oxaliplatin, *RT* radiotherapy, *AC* all-comers, *BD* biomarker driven

### Monotherapy

Pembrolizumab is a highly selective, humanized monoclonal antibody against PD-1 that is designed to block the interaction between PD-1 and its ligands, PD-L1 and PD-L2. MMR deficiency has been reported to occur in 5% to 10% of CCAs [106]. In a phase II trial of mismatch repair deficient (dMMR) non-colorectal gastrointestinal cancers, 17 patients were treated with pembrolizumab. Of four BTC patients, one had a complete response, one had a partial response, and one stable disease, all with durable responses [107]. In the follow-up study of 8 BTC patients with dMMR, 2 complete responses were achieved and 4 other patients were labeled with stable disease, resulting in an ORR of 25% [108]. In Keynote158, 22 BTC patients with dMMR and MSI-H were treated with pembrolizumab after progression on or intolerance to at least one line of standard therapy (NCT02628067). The study yielded an ORR of 34.3% with a median PFS and OS of 4.1 and 23.5 months, respectively. Treatment-related adverse events occurred in 151 patients (64.8%). Thirty-four patients (14.6%) had grade 3 to 5 treatment-related adverse events, therefore, exhibiting a safe profile for pembrolizumab [109]. However, in MSS, MMR proficient BTC, the ORR to ICI monotherapy appears much lower. KEYNOTE-028 (NCT02054806) is an ongoing, multi-cohort, phase Ib trial of pembrolizumab monotherapy for patients with PD-L1-positive advanced solid tumors, including PD-L1-positive BTC. Interim safety and efficacy data have been reported for a small cohort of patients with PD-L1-positive BTC. Twenty-four BTC patients with unknown MMR status were enrolled and showed an ORR of 13.0% with 3 partial responses, and the median PFS and OS were 1.8 and 6.2 months, respectively. The 12-month OS rate was 27.6% [110]. A larger BTC patient population with MSS is included in the Keynote-158 phase II trial to be treated. Among 104 patients, 6 patients had a confirmed partial response with an ORR of 5.8%. Surprisingly, one patient had a PD-L1 negative tumor. The median duration of response (DOR) was not reached and median PFS and OS were 2.0 and 7.4 months and 50% of responses were ongoing for at least 24 months [110]. Grade 3–5 treatment-related AEs occurred in 13.5% in Keynote158 (1 case of grade 5 immune-related renal failure) and 16.7% of patients in Keynote028 (no grade 5). 18.3% in Keynote158 and 20.8% of patients in Keynote028 had an immune-mediated AE or infusion reaction [110].

Nivolumab is a monoclonal antibody that binds to the PD-1 and blocks its interaction with PD-L1 and PD-L2. The results from a phase II trial in advanced refractory BTC showed an ORR of 11% with 5 MSS patients out of 46 achieving a partial response and DCR of 50% with durable responses lasting two years [111]. A phase I study with nivolumab, as monotherapy or combined with chemotherapy in 30 Japanese patients with BTC. One of 30 patients had an objective response in the monotherapy cohort with a median PFS and OS of 1.4 and 5.2 months, respectively [112].

Durvalumab is a human immunoglobulin G1 kappa (IgG1κ) monoclonal antibody that blocks the interaction of PD-L1 with the PD-1. In this phase I study preliminary results [113], the DCR was 16.7% at 12 weeks with durvalumab monotherapy. The median duration of response for durvalumab cohort was 9.7 months and median OS was 8.1 months. No unexpected toxicities were observed. Tremelimumab is a monoclonal antibody against CTLA-4. There are ongoing trials exploring the role of tremelimumab in solid tumors, including BTC (NCT01938612).

### Other immunotherapies

T cell based therapy is an emerging field and efficacy has been greatly appreciated in hematological malignancies [114] and melanoma [115]. This therapy for epithelial malignancies, such as BTC is under investigation. A successful case of metastatic BTC treated with mutation specific adoptive T-Cell therapy was reported. The autologous tumor infiltrating lymphocytes (TILs) that were specific to a mutated antigen ERBB2 interacting protein (ERBB2IP) expressed by the patient’s cancer were harvested and expanded in vitro. After lympho-depletive chemotherapy, the patient received the infusion of antigen-specific TILs and there was a significant reduction in the size of tumor lesions [116]. There are several ongoing trials in BTC involved with mesothelin-based immunotoxin LMB-100 (NCT04034238), mesothelin antibody anetumab ravtansine (NCT03102320), and mesothelin CAR-T therapy (NCT03907852).

### Combination therapy

Various studies in combination with different therapy modalities have been evaluated with the goal of improving the efficacy of ICI monotherapy (Table 4). Considering the multiple mechanisms adopted by tumor cells to evade the immune system through cancer immunoediting, the combination of ICIs and chemotherapy appears to be a promising strategy. It enhances the recognition and elimination of tumor cells by the host immune system, and reduces the immunosuppressive tumor microenvironment, while eradicating the tumor through DNA damage, inhibiting DNA replication, and preventing mitosis caused by cytotoxic chemotherapy [117]. A similar synergistic effect can be observed when combining ICIs with other treatments such as targeted therapies and radiation due to the immunomodulatory effects of targeted agents and radiation.

A phase I study involved with advanced BTC in Japanese population treated with nivolumab alone or in combination with GemCis showed that 11 of 30 patients had an objective response with a median PFS and OS of 4.2 and 15.4 months in the combination cohort [112]. In a phase I study of advanced BTC treated with the combination of durvalumab plus tremelimumab, preliminary results showed disease control rate was 32.2% at 12 weeks. The median duration of response was 8.5 months and median OS was 10.1 months [113]. Another phase I study reported the use of tremalimumab in combination with microwave ablation in the patients with advanced refractory BTC [118]. Two patients among sixteen evaluable patients achieved a confirmed partial response. Median PFS and OS was 3.4 and 6.0 months, respectively. An observational study reported the results of the use of lenvatinib plus nivolumab and pembrolizumab in 14 patients with iCCAs. The data showed ORR of 21.4% with 3 pts achieved partial response (PR) and DCR of 92.9%. Median PFS was 5.9 months. The most common adverse events included hypertension, aminotransferase elevation and fatigue [119]. Other ongoing trials with ICIs in combination with different regimens in advanced BTC are summarized in Table 4.

## Future directions

**Fig. 1:**
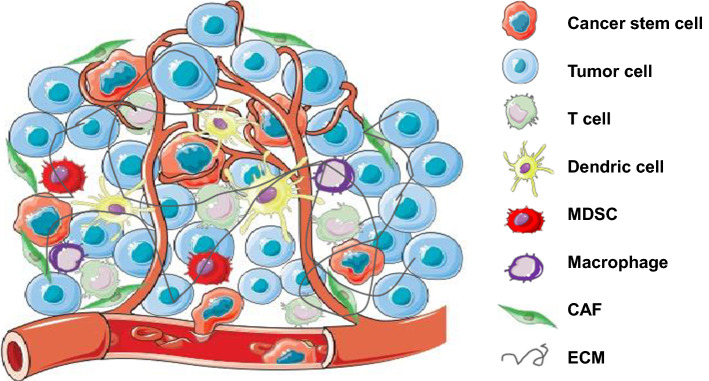
Tumor microenvironment of biliary tract cancer and potential cellular targets in combination with immunotherapy. There are different cell types that compose the immunosuppressive tumor microenvironment. It is a potential strategy to improve the efficacy of immune checkpoint inhibitors in combination with the agents targeting on other immune cells. *MDSC* myeloid derived suppressive cell, *CAF* cancer-associated fibroblast, *ECM* extracellular matrix

The systemic treatment strategies of advanced BTCs have been evolving for the last decade. There is established efficacy of the first-line chemotherapy GemCis and several potential triplet therapy regimens are under the evaluation. Still, no standard second-line chemotherapy regimen has been recommended though efforts have been made to test some candidates. The understanding of the molecular landscape of BTC has shed light on promising precision medicine with targeted therapy. Pemigatinib is approved for treatment of advanced refractory BTC with FGFR2 fusion with other rearrangement. The combination of chemotherapy and targeted agents has not shown enthusiastic outcomes. Nevertheless, several ongoing trials are assessing combination options. Recent breakthroughs of knowledge in cancer immunology have attracted a wave of immunotherapy options being investigated in cancers including BTC. However, the modest efficacy of single agent immunotherapy in advanced BTC is leading to various ongoing clinical trials using combinations of chemotherapy, targeted therapy, radiation therapy and other immunotherapy agents with the purpose of boosting the outcome of single agents. It is unclear the intratumorally immunological effect caused by targeted therapy or radiation therapy in BTC. Nevertheless, immunosuppressive tumor microenvironment composes of different immune cell population (Fig. 1) that needs intelligent design to identify the best therapeutic approaches to improve outcome. The increased numbers of available data from clinical trials concurrently opens more questions to be answered.

Since the patients included in the majority of clinical trials of BTC were heterogenous in terms of origin of diseases, this likely interferes with the interpretation of results from clinical trials no matter which treatment modalities have been applied. Efforts should be taken to enroll patients with specific origin of diseases or specific genetic backgrounds, however, that would certainly require global efforts to recruit enough patients. Fortunately, there are more trials being designed with this consideration, especially in iCCAs and GBC. With these efforts, we should be able to appreciate more significant results from homogenous patient populations and guide clinical practice with a disease location-specific approach. Furthermore, this heterogenicity is not only existing as disease per se, but the genetic background of individual patients is heterogenicity as indicated by mutation landscape and certainly contributes to the heterogenicity of the tumor microenvironment of BTCs as a whole. Furthermore, this plays a critical role in a patient’s response to immunotherapy. This heterogenicity has to be considered when the trials are designed, and when data are interpreted.

This notion leads to the other challenge, which is to stratify the subgroup of patients who will be more likely to respond to treatment. For example, there are multiple studies to explore the immune biomarkers at baseline from peripheral blood samples or biopsied tumor samples to stratify the likely responsible patients to immunotherapy, including tumor mutation burden, circulating tumor DNA, tumor infiltrating lymphocytes, PD-L1 expression in the tumor, etc. Currently, it is unclear if these markers would have predicative value in the immunotherapy of BTCs and it remains an important topic to be illuminated.

Lastly, most completed and ongoing trials with the combination strategies are involved with the administration of agents concurrently. It is unclear whether sequential treatment and maintenance therapy would be alternative and superior to concurrent approach. Additional investigation is needed to unravel these possibilities.
